# Alumni Meeting, Baltimore College of Dental Surgery

**Published:** 1880-03

**Authors:** 


*Alumni Meeting.*—The annual meeting of the Alumni Association of the Baltimore College of Dental Surgery was held in the College Building, on Thursday morning, March 4th, commencing at 10 o'clock.

A large number of Alumni were present, and great interest was manifested in the proceedings. The election of officers for the ensuing year resulted as follows:—President—Dr. S. J. Cockerille, of Washington, D. C. First Vice-President—Dr. W. H. Hoopes, of Baltimore. Second Vice-President—Dr. Wm. A. Mills, of Baltimore. Treasurer—Dr. A. W. Sweeny, Jr., of Baltimore. Secretary—Dr. L. M. Cowardin, of Richmond, Va.

The College Prizes were announced as follows:—For best clinical operator, Thos. M. Hunter, of Enfield, N. C. Prize: Set of Harris' Forceps, from S. S. White, Philadelphia. Honorable mention of J. Ryerson Smith, of Williston, S. C.

For best Thesis and Appliances for Correction of Irregularities of the Teeth. Louis C. F. Hugo, of Washington, D. C. Prize: Dental Engine, from S. S. White, of Philadelphia. Honorable mention of N. A. Hollinshead, of Fort Valley, Ga.

The best set of Artificial Teeth on metal base. Thomas M. Hunter, of Enfield, N. C. Prize: Set of Varney Pluggers, from S. S. White, Philadelphia.

For highest number of votes for final examinations. Thos. M. Hunter, of Enfield, N. C. Prize: Bartholow's Mat. Med. and Therapeutics, from Prof. Gorgas.

